# Diabetic retinopathy as a predictor of cardiovascular morbidity and mortality in subjects with type 2 diabetes

**DOI:** 10.3389/fmed.2022.945245

**Published:** 2022-08-16

**Authors:** Joan Barrot, Jordi Real, Bogdan Vlacho, Pedro Romero-Aroca, Rafael Simó, Didac Mauricio, Manel Mata-Cases, Esmeralda Castelblanco, Xavier Mundet-Tuduri, Josep Franch-Nadal

**Affiliations:** ^1^Primary Health Care Center Dr. Jordi Nadal i Fàbregas (Salt), Gerència d’Atenció Primària, Institut Català de la Salut, Girona, Spain; ^2^Diabetis des de l’Atenció Primária (DAP)-Cat Group, Unitat de Suport a la Recerca Barcelona, Fundació Institut Universitari per a la Recerca a l’Atenció Primària de Salut Jordi Gol i Gurina (IDIAPJGOL), Barcelona, Spain; ^3^Fundació Institut Universitari per a la Recerca a l’Atenció Primària de Salut Jordi Gol i Gorina (IDIAPJGOL), Barcelona, Spain; ^4^Departament of Medicine, Universitat Autònoma de Barcelona, Barcelona, Spain; ^5^Hospital de la Santa Creu i Sant Pau, Barcelona, Spain; ^6^Ophthalmology Service, University Hospital Sant Joan, Institut de Investigacio Sanitaria Pere Virgili (IISPV), University of Rovira and Virgili, Reus, Spain; ^7^Diabetes and Metabolism Research Unit, Department of Endocrinology, Vall d’Hebron University Hospital, Vall d’Hebron Research Institute, Barcelona, Spain; ^8^Department of Medicine, Faculty of Medicine, Autonomous University of Barcelona, Barcelona, Spain; ^9^Centro de Investigación Biomédica en Red de Diabetes y Enfermedades Metabólicas Asociadas (CIBERDEM), Barcelona, Spain; ^10^Department of Endocrinology and Nutrition, Hospital de la Santa Creu i Sant Pau, Barcelona, Spain; ^11^Departament of Medicine, University of Vic—Central University of Catalonia, Vic, Spain; ^12^Centre d’Atenció Primària La Mina, Gerència d’Àmbit d’Atenció Primària de Barcelona, Institut Català de la Salut, Barcelona, Spain; ^13^Division of Endocrinology, Metabolism and Lipid Research, John T. Milliken Department of Medicine, School of Medicine, Washington University in St. Louis, St. Louis, MO, United States; ^14^Primary Health Care Center Raval Sud, Gerència d’Àmbit d’Atenció Primaria, Institut Català de la Salut, Barcelona, Spain

**Keywords:** diabetic retinopathy, macrovascular complication, primary healthcare, real word data analyses, mortality

## Abstract

This study aimed to evaluate the predictive value of diabetic retinopathy (DR) and its stages with the incidence of major cardiovascular events and all-cause mortality in type 2 diabetes mellitus (T2DM) persons in our large primary healthcare database from Catalonia (Spain). A retrospective cohort study with pseudo-anonymized routinely collected health data from SIDIAP was conducted from 2008 to 2016. We calculated incidence rates of major cardiovascular events [coronary heart disease (CHD), stroke, or both—macrovascular events] and all-cause mortality for subjects with and without DR and for different stages of DR. The proportional hazards regression analysis was done to assess the probability of occurrence between DR and the study events. About 22,402 T2DM subjects with DR were identified in the database and 196,983 subjects without DR. During the follow-up period among the subjects with DR, we observed the highest incidence of all-cause mortally. In the second place were the macrovascular events among the subjects with DR. In the multivariable analysis, fully adjusted for DR, sex, age, body mass index (BMI), tobacco, duration of T2DM, an antiplatelet or antihypertensive drug, and HbA1c, we observed that subjects with any stage of DR had higher risks for all of the study events, except for stroke. We observed the highest probability of all-cause death events (adjusted hazard ratios, AHRs: 1.34, 95% CI: 1.28; 1.41). In conclusion, our results show that DR is related to CHD, macrovascular events, and all-cause mortality among persons with T2DM.

## Introduction

Diabetes mellitus has become one of the major public health challenges globally, both in developed and developing countries (1). The increasing prevalence of diabetes worldwide is caused by a complex interplay of socioeconomic, demographic, environmental, and genetic factors. The International Diabetes Federation (IDF) estimates that 10.5% of adults between 20 and 79 years have diabetes, equating to 537 million people (2). The IDF also estimates that 643 million adults will live with diabetes in 2030 (11.3% of the population), and the number will reach 783 million (12.2%) by 2045.

The main burden of diabetes results from its complications. Diabetic retinopathy (DR) is a microvascular and neurodegenerative complication whose prevalence increases with disease duration and causes a high risk of severe visual impairment and blindness (3, 4). According to recently published studies, there is considerable variability in the prevalence of DR. The meta-analysis of Yau et al. reports that one in every three subjects with diabetes will present some degree of DR (5) compared to 27% in the last review and the analysis of the IDF from the studies published in recent years (6).

Diabetes can cause numerous complications that weaken health, lower quality of life, and cause early death. People with diabetes are two to three times more likely to develop cardiovascular disease, and the risk of death doubles compared to people without diabetes (7).

The consequences of late detection of DR go beyond the resulting suboptimal visual acuity (8, 9). We have extensive evidence that associates DR with other micro- and macrovascular complications of diabetes. Recently, it was reported that DR is associated with subclinical atherosclerosis (10–14), macrovascular comorbidities such as coronary disease (15–20) and cerebrovascular accident (21–26). Of interest, some studies have reported an association between cognitive impairment and the incidence of dementia [risk ratio (RR), 1.3; 95% confidence interval (CI), 1.27–1.58] (27, 28), and the relation between DR and neurodegeneration diseases such as Parkinson’s disease has been proposed but remains unclear (29). In addition, a recent meta-analysis with observational studies found that subjects with diabetes who have DR have an increased risk of mortality from all causes compared to subjects with diabetes who do not have retinopathy (RR: 2.33, 95% CI: 1.92; 2.81) (30–32).

So far, to the best of our knowledge, there is a lack of studies evaluating the relation between DR and major cardiovascular events from primary healthcare settings. In this study, we aimed to evaluate the predictive value of DR, its severity with the incidence of major cardiovascular events [coronary heart disease (CHD) and stroke], and all-cause mortality in subjects with T2DM in a Mediterranean region.

## Materials and methods

The study included a retrospective cohort of subjects with T2DM from the SIDIAP database (Sistema de Información para el desarrollo de la Investigación en Atención Primaria). The SIDIAP database routinely collects pseudo-anonymized health data from users who attend the primary healthcare centers of the Catalonian Health Institute (Institut Català de la Salut, ICS). The ICS is the primary healthcare provider in Catalonia (Spain), covering about 80% (5,564,292 persons) of the Catalonian population. The SIDIAP database contains comprehensive patient data, such as visits with healthcare professionals, diagnoses, demographic information, clinical variables, laboratory test results, prescriptions, referrals to specialists and hospitals, and medication obtained from pharmacies. For this analysis, data were extracted covering a 10-year period. The inclusion period was defined from 1 January 2008 to 31 December 2012. The follow-up period was until the data extraction end date (31 December 2017) or a discontinuation event (death or any other database dropout).

### Participants

All subjects aged between 30 and 89 years with a diagnosis of T2DM, defined as the presence of International Classification of Diseases, 10th Revision (ICD-10) diagnostic codes: E11, E14 (and their subcodes), and screening for fundus photography (to determine the presence of DR), were included in the analysis. We excluded those subjects with other types of diabetes (type 1 diabetes, gestational diabetes, secondary, or other), without diagnostic codes for T2DM, subjects with cardiovascular events, and/or absence of fundus photography. The eligible participants were followed up for at least 5 years or until the discontinuation event.

### Variables

For all the included subjects, the presence or absence of DR was assessed using reports pertaining to digital 45° color fundus images. Two photographs were taken for each eye, the macula-centered and between the macula and the optic nerve. We only included fundus photography from patients screened at primary healthcare centers. DR was classified into different stages according to the Early Treatment Diabetic Retinopathy Study (ETDRS) classification (33) as no apparent retinopathy (NDR), mild non-proliferative retinopathy (NPDR), moderate NPDR, severe NPDR, proliferative diabetic retinopathy (PDR), and diabetic macular edema (DME). The DR diagnosis was taken from the worst-affected eye, and from the most recent photograph in case there was more than one screening during the inclusion period. Also, at inclusion, we collected socio-demographic variables (age, gender), toxic habits (current tobacco use), and clinical variables related to diabetes [age at diagnosis of diabetes, diabetes duration, and glycated hemoglobin levels (HbA1c)]. Data on cardiovascular risk factors [body mass index (BMI), blood lipids, total cholesterol, low-density lipoprotein (LDL) cholesterol, high-density lipoprotein (HDL) cholesterol, non-HDL cholesterol, blood pressure, pulse pressure] were collected. Additional data were gathered on medication. Obesity was defined as a BMI ≥ 30 kg/m^2^. The estimated glomerular filtration rate (eGFR) was calculated according to the CKD Epidemiology Collaboration (CKD-EPI) equation. Chronic kidney disease (CKD) was defined as an estimated glomerular filtration rate (eGFR) < 60 ml/min/1.73 m^2^ calculated using the CKD-EPI equation and/or a ratio of albumin/creatinine (CAC) in urine ≥ 30 mg/g.

During the follow-up period, we collected data on mortality by any cause and by severe cardiovascular events such as stroke (defined by ICD-10 codes and subcodes for “cerebral infarctions” and/or “transient cerebral ischemic attacks and related syndromes”) or CHD defined by ICD-10 codes and subcodes for “angina pectoris” and/or “acute myocardial infarction and/or subsequent myocardial infarction” and/or “certain current complications following acute myocardial infarction” and/or “chronic ischemic heart disease.” Additionally, we created a composite event as a combination of stroke and/or CHD, named as “macrovascular events.” The definition of the study variables and codes are summarized in Supplementary Table 1.

### Statistical analysis

A descriptive statistical analysis was carried out, summarizing the quantitative parameters with the mean and its standard deviation (SD), median or interquartile range, and the qualitative variables with frequency and percentage. We used an opportunistic sampling technique to capture all persons meeting the study inclusion criteria. To assess the association of the main study events considering the time of follow-up, a time-to-event complete case analysis (CCA) was performed, adjusting Cox proportional hazards models (34). Unadjusted hazard ratios (UnAHRs) and adjusted hazard ratios (AHRs), 95% CI, and *p*-value were estimated and summarized. We used Kaplan–Meier survival curves to graphically visualize the cumulative incidence for study events during the follow-up period in each group. Different study events were fully adjusted considering potentially confounding clinical variables such as DR, sex, age, BMI, tobacco, duration of T2DM, antiplatelet or antihypertensive drug treatment, and HbA1c. Additionally, we performed a model adding lipid-lowering and antidiabetic drugs to the previous model. To treat missing data, a multiple imputation analysis (MICE) was performed with the statistical package (35) using ten replicates and five iterations. The CCA and MICE analyses were compared in a sensitivity analysis, adjusting the models for different variables (Model 1: unadjusted; Model 2: adjusted for age and sex; Model 3: adjusted for sex, smoking, antiplatelet or antihypertensive drug treatment, and BMI; Model 4: fully adjusted). *P*-values less than 0.05 were considered statistically significant without using the correction for multiple comparisons for the multiple events analyzed. We used the cox.zph function in the survival package (R statistical software) to check the proportional hazards assumption of Cox models (34). Statistical analysis was performed with R statistical software, version 3.6.1.^1^

### Institutional review board statement

This study was approved by the Institutional Review Board (or Ethics Committee) of IDIAP Jordi Gol i Gurina Foundation (protocol code P13/028 and date of approval 03/04/2013).

## Results

**FIGURE 1:**
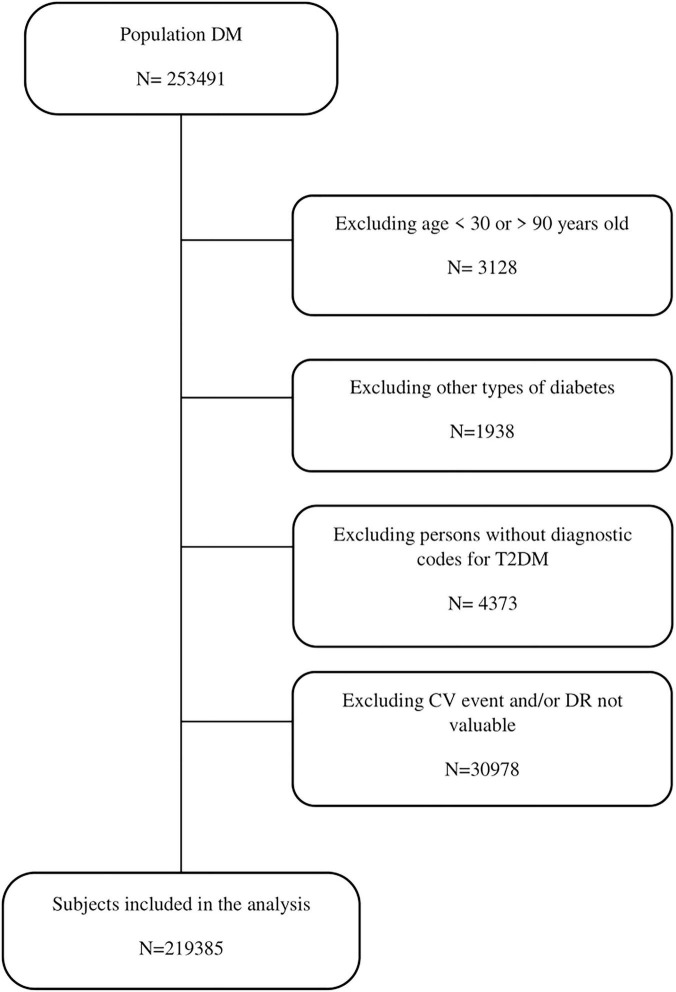
Study flow chart.

At the end of the inclusion period, 219,385 (86.5%) subjects met the study inclusion criteria and were included in the analysis. We identified 22,402 (10.2%) persons with any stage DR. Figure 1 shows the study flow diagram. The mean age (SD) of the subjects was 64.6 (± 11.6) years; the cohort contained a slightly higher proportion of men than women (55.7%) (Table 1).

**TABLE 1:** Clinical characteristics of the subjects at inclusion.

	All *N* = 219,385	Without diabetic retinopathy *N* = 196,983	With diabetic retinopathy *N* = 22,402
Male sex (%)	122,280 (55.7)	109,984 (55.8)*	12,296 (54.9)*
Age (years), mean (*SD*)	64.6 (11.6)	64.4 (11.5)*	67.0 (11.6)*
Current tobacco use (%)	58,602 (26.0)	52,839 (28.1)*	5,763 (26.9)*
**Clinical variables, mean (*SD*)**			
Diabetes duration (years)	5.18 (5.27)	4.84 (4.95)*	8.10 (6.81)*
BMI (kg/m^2^)	30.6 (5.22)	30.7 (5.20)*	30.2 (5.33)*
SBP (mmHg)	134 (14.8)	134 (14.5)*	137 (16.3)*
DBP (mmHg)	77.1 (9.59)	77.3 (9.51)*	76.0 (10.2)*
Heart rate (beat/min)	76.5 (12.3)	76.4 (12.2)*	77.4 (12.4)*
**Laboratory variables, mean (*SD*)**			
HbA1c (%)	7.18 (1.53)	7.12 (1.49)*	7.76 (1.75)*
Total cholesterol (mg/dL)	198 (40.3)	198 (40.1)*	192 (41.5)*
HDL cholesterol (mg/dL)	49.0 (12.8)	49.0 (12.8)*	49.5 (13.4)*
LDL cholesterol (mg/dL)	117 (33.8)	118 (33.7)*	112 (34.4)*
Triglycerides (mg/dL)	168 (122)	169 (123)*	160 (112)*
GFR (CKD-EPI; ml/min/1.73 m^2^)	78.0 (20.1)	78.8 (19.7)*	70.9 (22.4)*
Albumin-to-creatinine ratio	32.9 (131)	28.9 (114)*	67.5 (226)*
**Comorbidities (%)**			
Dyslipidemia	104,442 (47.6)	93,347 (47.4)*	11,095 (49.5)*
Hypertension	132,756 (60.5)	117,830 (59.8)*	14,926 (66.6)*
CKD	31,584 (14.4)	26,623 (13.5)*	4,961 (22.1)*
**Concomitant treatment (%)**			
Antihypertensive drugs	131,967 (60.2)	117,143 (59.5)*	14,824 (66.2)*
Antiplatelet drugs	52,396 (23.9)	44,581 (22.6)*	7,815 (34.9)*
Antidiabetics drugs	160,166 (73.0)	141,309 (71.9)*	18,857 (84.2)*
Lipid-lowering drugs	103,059 (47.0)	92,143 (46.8)*	10,916 (48.7)*

BMI, body mass index; CKD, chronic kidney disease; GFR, glomerular filtration rate; HbA1c, glycosylate hemoglobin; SD, standard deviation. *p < 0.001.

Compared to those with normal fundus photography, subjects who had DR (any stage) were older and had 1.6 times longer diabetes duration. Moreover, the DR group had a smaller proportion of smokers and a higher proportion (53.4%) of subjects with BMI < 30 kg/m^2^. Furthermore, the lipid profile was better in the DR group, with lower total cholesterol, triglycerides, and LDL cholesterol levels, and a higher proportion (28.3%) of subjects with LDL < 100 mg/dl and higher mean HDL (HDL cholesterol). Subjects with DR had a poorer renal profile than subjects without DR (eGFR, 70.9 (22.4) ml/min/1.73 m^2^ vs. 78.8 (19.7) ml/min/1.73 m^2^, respectively). The proportions of subjects (32.4%) with eGFR < 60 ml/min/1.73 m2 were higher than those without DR. We observed 1.6 times higher CKD prevalence among the subjects with DR than the subjects without DR. Regarding good glycemic control (HbA1c < 7%), statistically significant differences were observed among the groups, in favor of subjects without DR (61.3%) compared with subjects with DR (42.0%).

### Association between diabetic retinopathy and study events

Table 2 shows the epidemiology for different study events for both subjects with or without DR. During the follow-up period among the subjects with DR, mortality (all cause) was the most common event, followed by macrovascular events, CHD, and then stroke. The same pattern was observed among persons without DR, but with a lower incidence. The shortest time until the event was observed among the subjects with CHD and DR.

**TABLE 2:** The overall incidence for the study events between both groups.

Events	Groups of subjects	Patients/year	Event-free survival time (median in years)	Events (number)	Event rate (1,000 patients/year)
Mortality	No DR	1017964.6	4.950	17,527	17.2
	DR	112524.5	4.750	3,364	29.9
CHD	No DR	998687.0	4.846	6,360	6.3
	DR	109527.5	4.586	1,058	9.7
Stroke	No DR	1006859.7	4.893	3,637	3.6
	DR	110843.5	4.652	563	5.1
Macrovascular	No DR	988167.7	4.786	9,758	9.9
	DR	107933.7	4.498	1,584	14.7

CHD, coronary heart disease; stroke, macrovascular: CHD and/or stroke; event rate, incidence per 1,000 person-year.

In the bivariate analysis, we observed statistically significant, unadjusted hazard ratios for all of the events, with the risk for the study events being higher among the subjects with DR. When stratified for the stage of DR, the same tendency was observed. The highest risk for the study events was observed among the subjects with proliferative DR. We did not observe statistically significant HRs among the persons with DME and stroke events.

**FIGURE 2:**
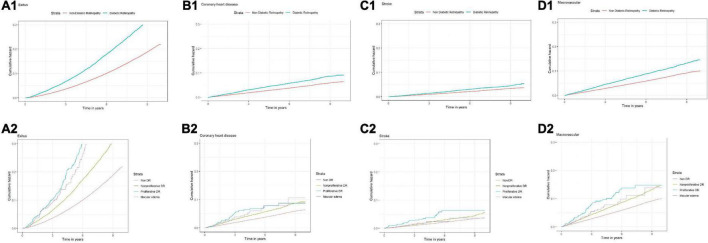
Survival curves and different study events and stages of diabetic retinopathy. **(A1)** All-cause mortality; **(B1)** coronary heart disease; **(C1)** stroke; **(D1)** macrovascular complications; **(A2)** all-cause mortality and different states of DR; **(B2)** coronary heart disease and different states of DR; **(C2)** stroke and different states of DR; **(D2)** and different states of DR and different states of DR.

In the multivariable CCA, fully adjusted for DR, sex, age, BMI, tobacco use, duration of T2DM, an antiplatelet or antihypertensive drug, and HbA1c, we observed that subjects with any stage of DR had higher risks for all of the study events. The probability of an event occurring was highest for all-cause death (AHR: 1.34, 95% CI: 1.28; 1.41) and lowest for stroke (AHR: 1.09, 95% CI: 0.97; 1.24). Similar results were observed when adding the lipid-lowering and antidiabetic drugs to the additional model. The Kaplan–Meier survival curves for study events are shown in Figure 2, and the results for the unadjusted and adjusted HR are summarized in Table 3.

**TABLE 3:** Hazard ratios for events among the study groups complete cases analysis.

	General		Stage of DR			
		
	Group without DR *N* = 196,983	Group with DR *N* = 22,402	Non-proliferative diabetic retinopathy (NPDR) *N* = 19,048	Proliferative diabetic retinopathy (PRD) *N* = 663	Diabetic macular edema (DME) *N* = 475	Unknown stage* *N* = 2,216
**Mortality, *n* (%)**	17,527 (8.90)	3,364 (15.0)	2,794 (14.7)	144 (21.7)	76 (16.0)	350 (15.8)
Unadjusted HR 95% CI [LL; UL]	Ref.	1.75 [1.69;1.82]	1.69 [1.62;1.76]	2.82 [2.39;3.32]	2.44 [1.95;3.06]	1.91 [1.72;2.12]
**Adjusted HR 95% CI [LL; UL]	Ref.	1.34 [1.28;1.41]	1.34 [1.27;1.42]	1.68 [1.31;2.15]	1.71 [1.27;2.31]	1.19 [1.03;1.38]
***Adjusted HR 95% CI [LL; UL]	Ref.	1.34 [1.27;1.41]	1.34 [1.26;1.41]	1.66 [1.30;2.13]	1.69 [1.25;2.28]	1.19 [1.03;1.38]
**CHD, *n* (%)**	6,360 (3.23)	1,058 (4.72)	904 (4.75)	38 (5.73)	22 (4.63)	94 (4.24)
Unadjusted HR 95% CI [LL; UL]	Ref.	1.84 [1.70;1.99]	1.51 [1.41;1.62]	2.00 [1.45;2.75]	1.82 [1.20;2.76]	1.39 [1.13; 1.70]
**Adjusted HR 95% CI [LL; UL]	Ref.	1.27 [1.16;1.39]	1.30 [1.19;1.43]	1.17 [0.71;1.92]	0.80 [0.38; 1.68]	1.11[0.86;1.44]
***Adjusted HR 95% CI [LL; UL]	Ref.	1.28 [1.17;1.40]	1.31 [1.19;1.44]	1.18 [0.72;1.92]	0.81 [0.38; 1.69]	1.11[0.86;1.44]
**Stroke, *n* (%)**	3,637 (1.85)	563 (2.51)	468 (2.46)	26 (3.92)	9 (1.89)	60 (2.71)
Unadjusted HR 95% CI [LL; UL]	Ref.	1.75 [1.64;1.86]	1.36 [1.24;1.50]	2.41 [1.64;3.55]	1.30 [0,68;2.50]	1.56 [1.20;2.01]
**Adjusted HR 95% CI [LL; UL]	Ref.	1.09 [0.97;1.24]	1.11 [0.97;1.26]	1.65[0.95;2.86]	0.74 [0.27;1.99]	0.93[0.65;1.33]
***Adjusted HR 95% CI [LL; UL]	Ref.	1.09 [0,97;1,24]	1.11 [0.97;1.26]	1.65[0.95;2.85]	0.74 [0.28;1.98]	0.93[0.65;1.33]
**Macrovascular, *n* (%)**	9,758 (4.95)	1,584 (7.07)	1,341 (7.04)	61 (9.02)	30 (6.32)	152 (6.86)
Unadjusted HR 95% CI [LL; UL]	Ref.	1.75 [1.64; 1.86]	1.47 [1.39; 1.55]	2.12 [1.65; 2.73]	1.62 [1.13; 2.31]	1.48 [1.26; 1.73]
**Adjusted HR 95% CI [LL; UL]	Ref.	1.22 [1.13; 1.31]	1.24 [1.15;1.34]	1.29 [0.88; 1.89]	0.79 [0.44; 1.44]	1.08 [0.87;1.33]
***Adjusted HR 95% CI [LL; UL]	Ref.	1.22 [1.14; 1.31]	1.25 [1.15;1.35]	1.30 [0.89; 1.90]	0.80 [0.44; 1.44]	1.08 [0.88;1.33]

DR, diabetic retinopathy; CHD, coronary heart disease; NDR, no apparent diabetic retinopathy; NPDR, non-proliferative diabetic retinopathy; PDR, proliferative diabetic retinopathy; DME, diabetic macular edema; ref, reference group; HR, hazard ratio; 95% CI, 95% confidence interval; LL, lower limit; UL, upper limit.

*Subjects having diabetic retinopathy by diagnostic code but without fundus photography/stage of DR.

**Adjusted for DR, sex, age, BMI, tobacco, duration of T2DM, an antiplatelet or antihypertensive drug, and HbA1c.

***Adjusted for DR, sex, age, BMI, tobacco, duration of T2DM, an antiplatelet, antihypertensive, lipid-lowering, or antidiabetic drugs, and HbA1c.

### Sensitivity analysis

**FIGURE 3:**
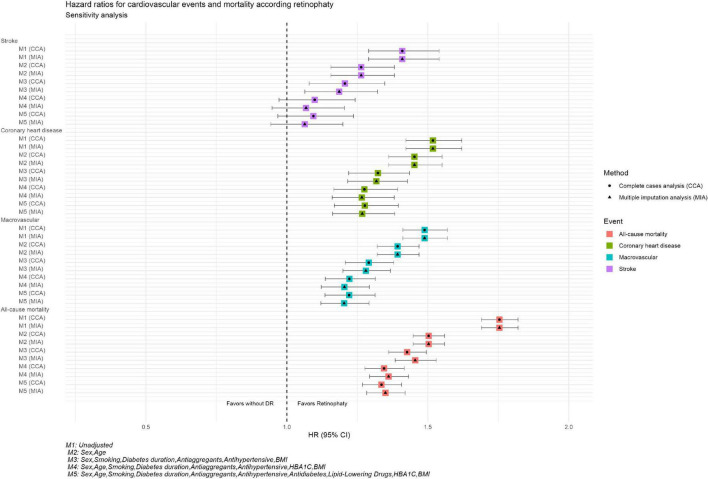
Hazard ratios for cardiovascular events and mortality according retinopathy Sensitivity analysis. M1, unadjusted; M2, sex, age; M3, sex, smoking, diabetes, antiaggregants, antihypertensive, BMI; M4, sex, age, smoking, diabetes, duration, antiaggregants, antihypertensive, HBA1C, BMI; M5, age, smoking, diabetes, duration, antiaggregants, antihypertensive, antidiabetes, libid-lowering drugs, HBA1C, BMI.

Figure 3 and Supplementary Table 2 show the HR observed in the sensitivity analysis. In the MICE, similar HRs for different study events were observed to those from the CCA. When comparing the two analyses for different adjusted models, we observed that HRs for the different events in the MICE analysis were going in the same direction as the CC analysis.

## Discussion

The results of our analysis with data collected from primary healthcare centers in Catalonia show a positive association between the DR and major cardiovascular events (CHD and stroke) and all-cause mortality.

Coronary heart disease, stroke, and DR have common risk factors, such as hyperglycemia, hyperlipidemia, and hypertension. Several studies have observed an association between DR and macrovascular disease and subclinical atherosclerosis (10–14). Some studies have reported that DR is a strong determinant of carotid intima-media thickness and arterial stiffness in patients with T2DM (12, 13). In the Atherosclerosis Risk in Communities (ARIC) cohort study based on a population of 1,524 people with T2DM without cardiovascular events at inclusion, during the follow-up of 7.8 years, the authors observed that the presence of DR was associated with the occurrence of CHD events (HR: 2.07, 95% CI: 1.38; 3.11) and fatal CHD (HR: 3.35, 95% CI: 1.40; 8.01) (16). Compared with the results observed in our study, during the follow-up over an average of 4.8 years, we observed lower but statistically significant adjusted risks for CHD (HR:1.27, 95% CI: 1.16; 1.39). In a recently reported systematic review and meta-analysis of cohort studies, including 17,611 patients without a previous history of CV disease at baseline, DR was associated with an increased risk of CV disease (relative risk (RR): 2.42, 95% CI: 1.77; 3.31) in diabetes (17). This risk was especially elevated among the T1DM persons (RR: 3.59, 95% CI: 1.79; 7.20) compared with T2DM persons (RR: 1.81, 95% CI: 1.47; 2.23) (17). Regarding the severity of DR, only a few studies have evaluated the association between the stage of DR and CV events. The studies done by Gimeno-Orna et al. and Targher et al. reported an increased risk between NPDR and RDP and the incidence of non-fatal or fatal cardiovascular events, independently of other known cardiovascular risk factors (HR:1.71, 95% CI: 1.1; 2.66 and HR: 2, 95% CI: 1.1; 3.56, respectively) (18, 19). Additionally, the Japan Diabetes Complications Study (JDCS) that included 2,033 subjects with T2DM with an 8-year follow-up period found an increased risk for CHD even during the initial stages of DR (mild-to-moderate NPDR) after adjusting for traditional CV risk factors (HR:1.69, 95% CI: 1.17; 2.97) (20). Our study observed a similar tendency but with a lower HR for NPDR and CHD in a fully adjusted model.

Evidence suggests that subjects with diabetes and DR seem to be at a high risk of ischemic stroke, with a meta-analysis that included 19 observational cohort studies of 81,452 diabetic patients reporting that the presence of DR was associated with an increased risk of stroke (HR: 1.25, 95% CI: 1.12; 1.39). Subgroup analysis for the type of diabetes yielded a pooled HR of 1.29 and 95% CI: 1.10; 1.50 in T2DM (21). In a secondary analysis of patients enrolled in the Action to Control Cardiovascular Risk in Diabetes (ACCORD) Eye Study in an adjusted Cox regression model, DR was independently associated with incident stroke (HR:1.52, 95% CI:1.05; 2.20) (22). Another prospective cohort study with 1,617 middle-aged people with diabetes observed that during a follow-up of 7.8 years, DR was associated with an increased risk of ischemic stroke (HR: 2.34, 95% CI: 1.13; 4.86) (24). In our study, we did not observe associations between DR or stage of DR and stroke in fully adjusted models, which could be due to the methodological differences or diagnostic codes used to identify the cases with stroke.

Regarding all-cause mortality, our study shows that the presence of any degree of DR is correlated with an increased risk of this event. In a study from the United States with 4,777 adults, the authors found that those persons with mild and moderate/severe retinopathy had an increased risk of all-cause mortality (HR: 1.81, 95% CI: 1.29; 2.55 and HR: 4.14, 95% CI: 1.77; 9.69, respectively) (30). In another meta-analysis from ten observational studies with 11,239 diabetic patients, the authors reported a doubling in the risk of mortality due to CVD in subjects with diabetes and severe DR (31). Similar results were also obtained in the analysis of other studies with similar follow-up periods in a meta-analysis of 19 studies encompassing 142,625 participants; the risk ratio (RR) for all-cause mortality with DR was 2.33 (95% CI: 1.92; 2.81) compared to subjects without DR. According to the different degrees of DR in subjects with T2DM, the RR of all-cause mortality varied. The RDNP risk was 1.38 (1.11–1.70), while the risk of PDR was 2.32 (1.75; 3.06) (32). Our analysis only observed statistically significant risks for all-cause mortality stratified for different stages of DR; this risk was highest among the persons who had PDR.

Our study has some potential limitations inherent in observational studies with routinely collected healthcare data. Firstly, missing data for the study variables is an important limitation. We used pseudo-anonymized routinely collected health data, where patients were visited as part of the regular healthcare surveillance. To test the effect of this limitation, we did a CCA and multiple imputations of missing data, and the results were consistent. Due to the database characteristics, there is a possibility of registering errors related to the diagnostic code for DR. Therefore, we used the fundus photography results and combined them with diagnostic codes. This limitation could also be applied to the study events. We used a wide spectrum of codes to define each study event. Another limitation is the numerical imbalance between the different groups due to the study design and lack of randomization. Moreover, we should acknowledge there is an inherent possibility of a certain risk of detection bias related to the frequency of medical visits. To minimize this bias, we designed the study to include only DM subjects who had at least one visit. We included the subjects in the study on the day of diabetic eye screening. Therefore, all the subjects were active users of the system. The main strength of our study was the large study population which increases the statistical power and external validity. Similarly, the results of our study confirm the results of previous studies.

In conclusion, our results show that DR is associated with an increased risk of cardiovascular disease and death among persons with T2DM. These findings indicate the importance of early screening, identification, and proper treatment of subjects with DR to reduce the risk of macrovascular disease and death. Further functional studies are needed to evaluate the biological background of these complications of diabetes and the potential use of stages of DR as an early marker for major adverse cardiovascular events.

## Data availability statement

The data analyzed in this study is subject to the following licenses/restrictions: Restrictions apply to the availability of some or all data generated or analyzed during this study because they were used under a license. The corresponding author will, on request, detail the restrictions and any conditions under which access to some data may be provided. Requests to access these datasets should be directed to XM-T, mundetx@gmail.com.

## Ethics statement

This study was approved by the Institutional Review Board (or Ethics Committee) of IDIAP Jordi Gol i Gurina Foundation (protocol code P13/028 and date of approval 03/04/2013). Written informed consent for participation was not required for this study in accordance with the national legislation and the institutional requirements.

## Author contributions

JB: conceptualization. XM-T: methodology and resources. JR: software, formal analysis, and data curation. JR, BV, and JB: validation. JB and BV: writing—original draft preparation and review and editing. DM, PR-A, RS, JR, EC, JB, MM-C, and JF-N: supervision. All authors have read and agreed to the published version of the manuscript.

## Footnotes

1
https://www.r-project.org
