# Supplementary material for: Diabetic retinopathy as a predictor of cardiovascular morbidity and mortality in subjects with type 2 diabetes
Source: Front Med (Lausanne). 2022 Aug 16;9:945245. doi: 10.3389/fmed.2022.945245 (PMC9424917; doi:10.3389/fmed.2022.945245)
Supplement: Supplementary file 1 [file Table_1.pdf]

Supplementary material

Diabetic retinopathy as a predictor of cardiovascular morbidity and mortality in subjects with type 2 diabetes

Joan Barrot-de la Puente, Jordi Real, Bogdan Vlacho, Pedro Romero-Aroca, Rafael Simó, Didac Mauricio, Manel Matas-Cases, Esmeralda Castelblanco, Xavier Mundet-Tudurí, Josep Franch Nadal

Supplementary Table 1	Page 1
Supplementary Table 2	Page 2

Supplementary Table 1. Code list

**Codes used for definition of the study variables**	
**Variable**	**Definition**
**Type 2 diabetes mellitus**	ICD-10-CM Codes: E11.xx; E14.xx
**Diabetic retinopathy**	ICD-10-CM Codes: E11.3; E14.3; H36; H36.0; H36.8 and/or fundus photography: mild non-proliferative retinopathy (NPDR), moderate NPDR, severe NPDR, proliferative diabetic retinopathy (PRD), and diabetic macular edema (DME)
**Dyslipidemia**	ICD-10-CM Codes: E78; E78.9 and/or Lipid-lowering drugs
**Hypertension**	ICD-10-CM Codes: 10 and/or Antihypertensive agents
**Macrovascular complications**	ICD-10-CM Codes: I21.xx; I22.xx; I23.xx; I25.xx; G45.xx; G46.xx; I63.xx;
**Coronary heart disease (CHD)**	ICD-10-CM Codes: I21.xx; I22.xx; I23.xx;
**Stroke**	ICD-10-CM Codes: G45.xx; G46.xx; I63.xx;
**Chronic kidney disease**	CKD-EPI glomerular filtration rate <60 ml/min/1,73m2 and or albumin/creatinine ratio>30mg/g
**Antithrombotic agents**	ATC/DDD codes: B01A
**Antihypertensive agents**	ATC/DDD codes: C02; C03; C07; C08; C09
**Antidiabetics agents**	ATC/DDD codes: A10
**Lipid-Lowering agents**	ATC/DDD codes: C10

xx: sub codes

Supplementary Table 2. Sensitivity analyses

Events	Variable	HR	95% lower limit	95% Upper limit	Method	Adjusted by
Mortality	Group with DR, ref (without DR)	1.75	1.69	1.82	CCA	Model 1
Mortality	Group with DR, ref (without DR)	1.75	1.69	1.82	MICA	Model 1
Mortality	Group with DR, ref (without DR)	1.50	1.44	1.55	CCA	Model 2
Mortality	Group with DR, ref (without DR)	1.50	1.44	1.55	MICA	Model 2
Mortality	Group with DR, ref (without DR)	1.42	1.36	1.49	CCA	Model 3
Mortality	Group with DR, ref (without DR)	1.45	1.38	1.52	MICA	Model 3
Mortality	Group with DR, ref (without DR)	1.34	1.27	1.41	CCA	Model 4
Mortality	Group with DR, ref (without DR)	1.36	1.29	1.43	MICA	Model 4
CHD	Group with DR, ref (without DR)	1.51	1.42	1.62	CCA	Model 1
CHD	Group with DR, ref (without DR)	1.51	1.42	1.62	MICA	Model 1
CHD	Group with DR, ref (without DR)	1.45	1.36	1.55	CCA	Model 2
CHD	Group with DR, ref (without DR)	1.45	1.36	1.55	MICA	Model 2
CHD	Group with DR, ref (without DR)	1.32	1.21	1.43	CCA	Model 3
CHD	Group with DR, ref (without DR)	1.31	1.21	1.42	MICA	Model 3
CHD	Group with DR, ref (without DR)	1.27	1.16	1.39	CCA	Model 4
CHD	Group with DR, ref (without DR)	1.26	1.16	1.38	MICA	Model 4
Stroke	Group with DR, ref (without DR)	1.40	1.28	1.54	CCA	Model 1
Stroke	Group with DR, ref (without DR)	1.40	1.28	1.54	MICA	Model 1
Stroke	Group with DR, ref (without DR)	1.26	1.15	1.38	CCA	Model 2
Stroke	Group with DR, ref (without DR)	1.26	1.15	1.38	MICA	Model 2
Stroke	Group with DR, ref (without DR)	1.20	1.07	1.34	CCA	Model 3
Stroke	Group with DR, ref (without DR)	1.18	1.06	1.32	MICA	Model 3
Stroke	Group with DR, ref (without DR)	1.09	0.97	1.24	CCA	Model 4
Stroke	Group with DR, ref (without DR)	1.06	0.94	1.20	MICA	Model 4

Macrovascular	Group with DR, ref (without DR)	1.48	1.41	1.56	CCA	Model 1
Macrovascular	Group with DR, ref (without DR)	1.48	1.41	1.56	MICA	Model 1
Macrovascular	Group with DR, ref (without DR)	1.39	1.32	1.46	CCA	Model 2
Macrovascular	Group with DR, ref (without DR)	1.39	1.32	1.46	MICA	Model 2
Macrovascular	Group with DR, ref (without DR)	1.29	1.20	1.37	CCA	Model 3
Macrovascular	Group with DR, ref (without DR)	1.28	1.19	1.36	MICA	Model 3
Macrovascular	Group with DR, ref (without DR)	1.22	1.13	1.31	CCA	Model 4
Macrovascular	Group with DR, ref (without DR)	1.20	1.12	1.29	MICA	Model 4

DR: diabetic retinopathy, CHD: Coronary Heart Disease; CCA: complete case analysis; MICA: multiple imputation analysis; ref: reference group; HR: hazard ratio;
Model1: un-adjusted;
Model 2: adjusted for age and sex;
Model 3: adjusted for sex, smoking, antiplatelet or antihypertensive drug treatment and BMI;
Model 4: fully adjusted (sex, age, BMI, Tabaco, duration of T2DM, antiplatelet or antihypertensive drug, and HbA1c)
